# Highly Accurate Diagnosis of Pleural Tuberculosis by Immunological Analysis of the Pleural Effusion

**DOI:** 10.1371/journal.pone.0030324

**Published:** 2012-01-25

**Authors:** Jayne S. Sutherland, Danlani Garba, Augustin E. Fombah, Awa Mendy-Gomez, Francis S. Mendy, Martin Antonio, John Townend, Readon C. Ideh, Tumani Corrah, Martin O. C. Ota

**Affiliations:** Medical Research Council Unit, Atlantic Road, Fajara, The Gambia, West Africa; Statens Serum Institute, Denmark

## Abstract

Pleural TB is notoriously difficult to diagnose due to its paucibacillary nature yet it is the most common cause of pleural effusions in TB endemic countries such as The Gambia. We identified both cellular and soluble biomarkers in the pleural fluid that allowed highly accurate diagnosis of pleural TB compared to peripheral blood markers. Multi-plex cytokine analysis on unstimulated pleural fluid showed that IP-10 resulted in a positive likelihood ratio (LR) of 9.6 versus 2.8 for IFN-γ; a combination of IP-10, IL-6 and IL-10 resulted in an AUC of 0.96 and positive LR of 10. A striking finding was the significantly higher proportion of PPD-specific IFN-γ+TNF-α+ cell population (PPD-IGTA) in the pleural fluid compared to peripheral blood of TB subjects. Presence of this pleural PPD-IGTA population resulted in 95% correct classification of pleural TB disease with a sensitivity of 95% and specificity of 100%. These data suggest that analysis of the site of infection provides superior diagnostic accuracy compared to peripheral blood for pleural TB, likely due to the sequestration of effector cells at this acute stage of disease.

## Introduction

Tuberculosis (TB) still remains one of the top three deadly diseases in developing countries with 1.7 million deaths recorded in 2009 [1]. While effective treatment is available, this relies on early and accurate diagnosis. To date, sputum smear (which is unreliable, particularly in HIV-positive and extrapulmonary TB) and sputum culture (which is time-consuming and expensive) remain the ‘gold-standard’ techniques for diagnosis of TB. Development of point-of-care diagnostics will have vast public health benefits by decreasing morbidity and mortality and reducing transmission rates of TB but this requires determination of biomarkers with high specificity and sensitivity for active TB disease.

Tuberculosis is the most common cause of pleural effusion in developing countries but it is notoriously difficult to diagnose due to its paucibacillary nature: positive cultures are seen in less than 25% of HIV-negative cases but can rise to 75% with HIV-infection [2]. Host immune factors provide greater diagnostic accuracy, including levels of IFN-γ and Adenosine Deaminase (ADA) [3], both of which have >95% specificity and sensitivity, appear to be unaffected by HIV co-infection and do not require any sample preparation [3]–[7]. However these markers are less definitive in TB-endemic countries and have not been evaluated in a West African cohort.

Tuberculosis infection occurs by inhalation of *Mycobacterium tuberculosis* (MTb) bacilli. These reside within lung macrophages and can be contained (but rarely eliminated) at early stages of infection: thus close to 2 billion people world-wide are infected with MTb and 10% of these will progress to active disease in their lifetime [1]. Progression to active disease occurs due to changes in bacterial virulence and/or the host immune system: the prime example shown with con-current HIV infection, which is the most potent known risk factor for progression to active tuberculosis [8]. The fact that the localised immune response is occurring in the lung suggests that analysis of the peripheral blood may not be representative of the host immune response, particularly at the acute stage of disease. Indeed, there is extensive literature showing sequestration of immune cells to the lung during TB [9]–[13], although their role appears to depend on the type and stage of disease [13].

In this study we analysed paired blood and pleural fluid samples from patients presenting with pleural effusion who were subsequently classified as having TB or not (malignancy, pneumonia, liver disease). The cellular and soluble profiles of the pleural fluid easily discriminated between subjects with TB and those without and should be further validated as tools for new and improved TB diagnostics.

## Methods

### Ethics statement

This study was conducted according to the principles expressed in the Declaration of Helsinki. Ethical approval was obtained from the Gambia Government/Medical Research Council (MRC) Joint Ethics Committee. All patients provided written informed consent for the collection of samples and subsequent analysis.

### Subjects

Subjects were included in this study if they were above 15 years of age and were seen at the TB clinic, general outpatient's clinic or admitted to the ward at MRC, Fajara with evidence of pleural effusion demonstrated by x-ray. Following informed consent, 50 mL of pleural fluid (PF) and 5 mL of peripheral blood (PB) was taken for immunological analysis. Pleural fluid and sputum (where possible) were sent to the microbiology lab for routine smear and Bactec culture. A sample of pleural fluid was also sent to the biochemistry lab for assessment of glucose and protein levels. HIV testing was performed on all consenting patients. We also performed Mantoux skin test reactivity (2 IU PPD, SSI, Denmark). 200 µL of PB was used to obtain differential cell counts using a Medonic haematology analyser (Merck).

### Classification of subjects presenting with pleural effusion

Subjects were classified as having TB if they had an exudative effusion (for this study, exudative effusion was classified as pleural protein >30 g/L and glucose <4 mM) and were bacteriologically culture positive (definite TB) or had a good response to TB treatment and had ADA levels >35 IU/mL and a lymphocytic infiltrate (>75% of PF cells) (probable TB; **Table 1**). Out of 41 subjects with evaluable data, 7 were confirmed TB by bacterial culture; 23 were classified as having probable TB and 11 were classified as ‘not TB’. Of these, 3 had liver disease (cirrhosis), 5 had pneumonia (*Streptococcus pneumoniae*), 2 had a malignancy and 1 was unclassified. Two subjects (19 and 21) had extremely high ADA levels; previously been shown to be indicative of empyema [7]. Subject 19 was however culture positive and thus classified as having TB disease while subject 21 was diagnosed radiologically and clinically with pneumonia. Subject 26 had extremely low ADA levels and had a final classification of Cirrhosis. While most of the effusions were lymphocytic, subject 12 had the lowest percentage (15%) and was diagnosed with pneumonia. This patient also had a high peripheral blood WBC count (10.8E^9^/L) with a granulocyte to lymphocyte ratio of 8.3, which we have previously shown to be indicative of pneumonia rather than TB [14]. 9 subjects were HIV-positive of which 7 were subjects with a classification of TB (4 were culture positive = definite TB and 3 were classified according to ADA levels and response to treatment = probable TB) and 2 with a classification of pneumonia. Subject 6 was Mtb culture positive and had immune reconstitution inflammatory syndrome (IRIS) after initiation of anti-retroviral treatment.

**Table 1:** Subject information and classification.

Subject	PF ADA	PF %L	Culture	Exudate	TST	HIV	Tx Response	Classification
1	n/d	88	neg	yes	pos	neg	improved	Probable TB
2	55	87	neg	yes	pos	neg	improved	Probable TB
3	39	95	neg	yes	n/d	neg	improved	Probable TB
4	51	81	neg	yes	pos	neg	improved	Probable TB
5	110	45	neg	yes	neg	pos	not given	Pneumonia
6	41	91	pos	yes	n/d	pos	improved	Definite TB
7	47	86	neg	yes	pos	neg	improved	Probable TB
8	45	73	neg	yes	pos	neg	improved	Probable TB
9	24	89	neg	yes	n/d	neg	not given	Cirrhosis
10	59	36	pos	yes	neg	pos	died	Definite TB
11	29	15	neg	yes	n/d	neg	died	Pneumonia
12	45	94	neg	yes	pos	neg	improved	Probable TB
13	58	n/d	neg	yes	neg	pos	improved	Probable TB
14	33	86	pos	yes	pos	pos	improved	Definite TB
15	45	93	n/d	yes	pos	neg	improved	Probable TB
16	29	94	neg	yes	neg	neg	died	Probable TB
17	16	77	n/d	yes	neg	neg	improved	Probable TB
18	58	62	pos	yes	n/d	neg	improved	Definite TB
19	689	65	pos	yes	pos	neg	improved	Definite TB
20	52	34	pos	yes	pos	neg	improved	Definite TB
21	1463	40	neg	yes	neg	neg	not given	Pneumonia
22	38	46	neg	yes	pos	neg	improved	Probable TB
23	n/d	39	neg	yes	neg	n/d	not given	Malignancy
24	39	84	neg	yes	neg	pos	improved	Probable TB
25	26	91	neg	yes	pos	neg	improved	Probable TB
26	0.82	84	neg	no	neg	neg	not given	Cirrhosis
27	45	83	neg	yes	pos	neg	improved	Probable TB
28	37	93	neg	yes	pos	neg	not given	Pneumonia
29	15	28	neg	yes	neg	neg	died	Probable TB
30	47	85	neg	yes	pos	pos	not given	Pneumonia
31	9	91	n/d	yes	neg	neg	not given	Malignancy
32	57	71	neg	yes	n/d	neg	improved	Probable TB
33	30	85	neg	yes	n/d	neg	not given	Unknown
34	6	88	neg	yes	neg	neg	improved	Probable TB
35	71	82	pos	yes	neg	pos	improved	Definite TB
36	39	n/d	neg	yes	pos	neg	improved	Probable TB
37	54	96	neg	yes	pos	neg	improved	Probable TB
38	59	34	neg	yes	neg	pos	Improved	Probable TB
39	0	n/d	neg	no	neg	n/d	not given	Cirrhosis
40	24	74	neg	yes	pos	neg	improved	Probable TB
41	68	85	n/d	yes	neg	neg	Improved	Probable TB

ADA = Adenosine Deaminase; n/d = not done; TST = Tuberculin skin test; PF = pleural fluid; %L = % lymphocytes; Exudate = PF protein >30 g/L and glucose >3.3 mmol/L; pos = positive; neg = negative; HIV = Human Immunodeficiency Virus; Culture = bacteriological culture of sputum or pleural fluid; Tx = treatment.

### Pleural fluid processing

Fresh PF samples were centrifuged at 600_gmax_ for 5 min. at room temperature (RT). 2×1.5 mL aliquots of fluid were stored at −20°C for later analysis of ADA and cytokine levels. The remaining cell pellet was resuspended in 5 mL of RPMI+10% FCS from which cell number and viability were determined using Trypan blue exclusion.

### Flow Cytometry

Approximately 1 million PF cells or 200 µL of PB was used for each test of a flow cytometry panel which all contained anti-FcR block at a final concentration of 1∶10. Ex vivo staining involved incubation with 20 µL of previously titrated antibody cocktails. Antibodies were purchased from Becton-Dickinson (USA) unless otherwise stated and included CD4-APC-Al750 (APCA; ebioscience, UK); CD8-Pacific Blue (PB), CD45RO-PECy5 (PC5), CD28-FITC, CD27-APC, CCR7-PE and CD62L-PECy7 (PC7; both from ebioscience, UK) for detection of naïve and memory T cell phenotypes; Vα24-FITC, Vβ11-PE (both from Dako, Denmark), CD4-PerCP (PCP), CD127 Allexa-647 (ebioscience, UK), CD25-PC7 (ebioscience, UK) and CD8-PB for detection of T regulatory cell phenotypes; CD209-FITC (ebioscience, UK), HLA-DR-PE, CD11c-PC5, CD14-APC, CD123-PC7 (ebioscience, UK), CD11b-APCCy7 (ebioscience, UK) and CD8-PB for detection of innate cells; γδTCR-APC, αβTCR-FITC, CD3-PC7, CD8-PB, CD4-PerCP, CD56-PE and CD19-APCA (ebioscience, UK) for detection of αβ T cells, γδ T cells, B and NK cells and Ki67-FITC, Perforin-PE, CD38-PC7 (ebioscience, UK), CD4-PCP, CD56-APC and CD8-PB for analysis of the functional status of the *ex vivo* cells. After 30 min. incubation at RT in the dark, 2 mL of FACS lysing buffer was added (Becton-Dickinson, USA) and cells incubated for a further 9 minutes. Tubes were then centrifuged (600_gmax_, 5 minutes), supernatant poured off, 1× wash in FACS buffer (PBS/FCS/Az) performed and finally resuspended in 300 µL of FACS buffer for flow cytometry acquisition.

### Intracellular cytokine analysis

#### Overnight antigenic stimulation

One million PF cells in 200 µL or 200 µL of PB was used for each test of an intracellular cytokine assay. Cells were stimulated with ESAT-6/CFP-10 fusion protein (10 µg/mL), PPD (10 µg/mL), or anti-CD3 (10 µg/mL). The negative control tube was incubated without antigen. Co-stimulation with anti-CD28 and anti-CD49d (Becton-Dickinson, USA) was added to all tubes (final concentration 2 µg/mL each). Tubes were vortexed and incubated for 2 hours. Brefeldin A was then added (final concentration 10 µg/mL), tubes vortexed, covered and incubated overnight (16 h) at 37°C, 5% CO_2_.

### Antibody staining

20 µL of antibody cocktail (CD45RO-PC5, CD27-FITC, CD8-PB (all from Becton-Dickinson, USA) and CD4-APCAl750 (ebioscience, UK) was added to each tube and incubated for 30 min at RT, in the dark. Red blood cells were lysed by addition of 2 mL FACS lysing buffer for 9 min followed by centrifugation at 600_gmax_ for 5 min. Supernatant was poured off and cells resuspended in 500 µL of 1X FACS Perm 2 solution (Becton-Dickinson, USA). Tubes were vortexed and incubated for a further 20 min, at RT, in the dark. Tubes were then centrifuged at 620_gmax_, supernatant removed and 20 µL of cytokine cocktail (TNF-α-PC7 at 1∶80 (ebioscience, UK), IFN-γ-APC at 1∶40 and IL2-PE at 1∶80 (both from Becton-Dickinson, USA) added. Cells were incubated for 30 min, RT, in the dark, washed by addition of 1 mL FACS buffer and centrifuged at 620_gmax_ for 5 min. Finally, cells were resuspended in 300 µL FACS buffer for acquisition.

### Flow cytometry acquisition and analysis

Cells were acquired using a CyAn ADP™ 9-colour flow cytometer (Beckman Coulter, USA). Lymphocytes were gated according to 90° FSC and SSC and compensation performed. For *ex vivo* staining, 100,000 lymphocytes were acquired and 200–500,000 were acquired for intracellular cytokine staining. Analysis was performed using FlowJo software version 9.2 (Treestar, USA). Analysis and presentation of distributions was performed using SPICE version 5.1, downloaded from http://exon.niaid.nih.gov/spice [15]. Comparison of distributions was performed using a Student's T test and a partial permutation test as described [15].

### Adenosine Deaminase (ADA) assay

To assess ADA levels in the pleural fluid we adapted the standard Galanti and Giusti protocol [7] according to the manufacturer's instructions (Diazyme, USA). Briefly, the high control provided with the kit was serially diluted to form a standard curve ranging from 145.2±24.7 to 4.5±0.8 IU/L. 5 µL of samples, standards, low control (31.4±5.3 IU/L) and calibrator (50 IU/L) were added to 180 µL of R1 (PNP) in duplicate in a flat-bottom 96-well plate (Nunc, Germany). Samples were incubated at 37°C for 5 min then 90 µL of R2 (XOD) was added to each well. Samples were incubated at RT and read on an ELISA plate reader (Multiscan, Labsystems, Finland) at 550 nm after 3 min. Data were analysed using Softmax Pro software (Molecular Devices, USA). A standard curve was constructed using the diluted high standard, the calibrator concentration was determined and results were adjusted accordingly. All samples were read in duplicate and mean values (IU/L) determined. This assay is specific for ADA, has no detectable reaction with other nucleosides and is not affected by serum bilirubin up to 20 mg/dL, hemoglobin up to 200 mg/dL, triglycerides up to 750 mg/dL, or ascorbic acid up to 4 mg/dL. Levels greater than 35 IU/L are indicative of TB in lymphocyte-predominant pleural fluid [7].

### 27-plex cytokine analysis of unstimulated pleural fluid

A 27-plex cytokine-bead kit was used (Bio-Rad, USA) and the assay performed according to the manufacturer's instructions. Cytokines, chemokines and growth factors assessed were: IL-1β, IL-1ra, IL-2, IL-4, IL-5, IL-6, IL-7, IL-8, IL-0, IL-10, IL-12(p70), IL-13, IL-15, IL-17, Eotaxin, FGF, G-CSF, GM-CSF, IFN-γ, IP-10, MCP-1, MIP-1α, MIP-1β, PDGF-BB, RANTES, TNF-α and VEGF. Following pre-wetting of the filter plate, 50 µl of bead suspension was added to each well and washed twice. 50 µl of samples and standards were then added, the plate was sealed and shaken for 30 sec at 1100 rpm, and incubated for 30 min at 300 rpm. The plate was washed 3 times then 25 µl of pre-diluted detection antibody was added. Following shaking, the plate was incubated for 30 min at 300 rpm in the dark. After washing, 50 µl of 1X streptavidin-PE was added to each well and incubated for 10 min. The plate was again washed and resuspended in 125 µl of assay buffer, sealed, mixed and immediately read on the Bioplex analyser using Bioplex manager software (version 4.0; Bio-Rad, USA) and a low PMT setting. All standards were run in duplicate.

### Statistical analyses

Data were analysed by Wilcoxon matched pairs test, Kruskal-Wallis test with Dunn's post-test comparison, Mann-Whitney U-test or multiple logistic regression analysis. Immunological analyses were performed on all subjects (HIV-positive and negative) and also on HIV-negative alone and we found no statistical difference. As such, all subjects are grouped together unless otherwise indicated. Furthermore, following validation with the TB-definite subjects, all subjects with probable or definite TB were grouped together for statistical purposes.

## Results

### The immune cell profile of the peripheral blood is significantly different to the site of infection in pleural TB

**Figure 1:**
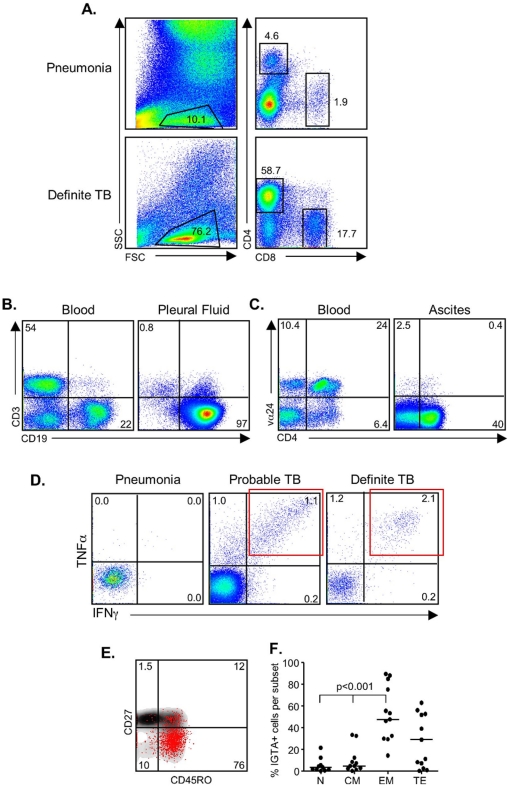
Flow cytometry comparison between peripheral blood and pleural fluid. (**a**) *Ex vivo* assessment of the cell phenotypes within the pleural fluid. Shown are representative flow cytometry profiles from a patient with pneumonia and one who was diagnosed with TB. Profiles are first gated on the lymphocyte population as determined by FSC and SSC. Following singlet and CD3 gating, analysis of CD4+ and CD8+ T cell populations is performed. (**b**) Analysis of a subject with pleural effusion caused by malignancy. In this case CD3 and CD19 are plotted together to illustrate T and B cell proportions in the blood (left) and pleural fluid (right) of a subject with a malignancy (subject 33). (**c**) Flow cytometry profile from blood and ascites where the blood had a distinct Vα24+CD4+ T cell population; absent from the ascites. (**d**) Functional analysis of PF cells. Overnight stimulation of PF cells with PPD was followed by intracellular cytokine detection. Following gating on the CD4+ T cells, TNF-α and IFN-γ positive cells were assessed. There was no response from the patient with a bacterial infection but a striking response from the patients with definite and probable TB. In particular, note the presence of a distinct IFN-γ+TNF-α+ double-positive cell population (IGTA; red box). (**e**) The CD4+ IGTA population was gated and the naïve/memory phenotype determined by CD27 and CD45RO expression (red-dot overlay). (**f**) Analysis of 12 subjects with evaluable data was performed using a Kruskal-Wallis test followed by Dunn's post-test comparison. We found the majority of the IGTA+ cells were of an effector memory phenotype (EM; CD27−CD45RO+), bottom right quadrant of (**d**). N = naïve (CD27+CD45RO−); CM = central memory (CD27+CD45RO+); EM = effector memory (CD27−CD45RO+) and TE = terminal effectors (CD27−CD45RO−).

We analysed the cellular content of the PF and compared this to the PB for each subject. Analysis of the lymphocyte and CD4+ T cell proportions in the PF indicated a likelihood of TB (**Fig. 1a**). Subject 11 was classified with *Streptococcus Pneumoniae* infection and had a granulocytic effusion (90%) whereas Subject 6, classified with TB (culture-confirmed) had a lymphocytic infiltrate. Furthermore, only 4.6% of the lymphocytes were CD4+ in Subject 11 compared to 58.7% in Subject 6 (**Fig. 1a**). Flow cytometry also allowed us to determine other underlying causes of the effusion, particularly malignancy and showed distinct differences between the blood and PF profiles. For example, assessment of the lymphocyte population for subject 33 showed normal blood levels of CD3+ T cells but elevated B cell levels (22%; **Fig. 1b**) and in the pleural fluid they had only 0.8% CD3+ T cells but 97% B cells (**Fig. 1b**). Flow cytometry of an ascites sample was also performed although this was not included in the statistical analysis. The ascites profile again was very distinct to that seen in the peripheral blood. The blood had a large Vα24+CD4+ proportion which was not present in the ascites (**Fig. 1c**). This patient was subsequently confirmed to have both TB and lymphoma.

Comparison between the PB and the PF for each patient was performed using a Wilcoxon matched-pairs test (**Table 2**). There was a significantly higher proportion of CD3+ T cells in the PF compared to the PB (median[IQR] = 72[43–80] for PF and 44[33–51] for PB; p = 0.0045) which was due to an increase in CD4+ T cells but not CD8+ T cells (p = 0.0001 for CD4+ T cells; **Table 2**), resulting in an increased ratio of CD4∶CD8 T cells (p = 0.0001). Further classification of the CD4+ T cells based on CD27 and CD45RO expression showed a significant decrease in the proportion of naïve cells (CD27+CD45RO−) but increase in central memory (CM) cells (CD27+CD45RO+) (p = 0.0004 and p = 0.0166 respectively; **Table 2**) in the PF compared to PB. Although the overall CD8+ T cell proportion was similar between the PF and PB, there was a significantly higher proportion of naïve CD8+ cells and lower proportion of CD8+ terminal effector (TE; CD27−CD45RO−) cells (p = 0.0173 and p = 0.0005 respectively; **Table 2**) in the PF. There was also an increased expression of CD127 (IL7Ra) on both the CD4+ and CD8+ cells (p = 0.0175 and p = 0.0005 respectively) in the PF compared to the PB. Increased expression of the activation marker, CD38, was also seen on the CD4+ T cells in the PF compared to the PB (median[IQR] = 6.3[4.0–15] for PF compared to 2.2[1.4–9.4] for PB (p = 0.0143; **Table 2**).

**Table 2:** Comparison of immune subsets in peripheral blood and pleural fluid of TB and non-TB patients.

	TB (n = 30)		non-TB (n = 11)		TB PF vs PB	PF TB vs no TB
Subset (%)	PB	PF	PB	PF	p-value	p-value
**CD3**	44[33–51]	72[43–80]	36[7.3–52]	64[1.2–82]	**0.0045**	0.7168
**CD4**	26[21–33]	45[33–61]	22[15–27]	34[2.7–63]	**0.0001**	0.2147
**CD8**	16[11–24]	17[12–23]	8.4[4.8–17]	10[0.9–16]	0.8092	**0.0099**
**4/8 ratio**	1.5[0.9–2.1]	3.4[1.5–5.0]	2.7[1.6–4.8]	2.4[1.4–5.6]	**0.0001**	0.7145
**CD19**	8.2[2.5–12.4]	3.9[1.1–10]	4.6[1.2–13]	6.0[2.3–7.3]	0.0691	0.8057
**CD56**	8.6[4.6–13]	2.5[1.6–4.6]	5.3[3.5–10]	1.0[0.1–4.7]	**0.0001**	0.0813
**γδTCR**	3.0[0.8–3.7]	1.5[1.0–2.8]	1.8[1.2–6.6]	2.1[0.4–3.0]	0.5230	0.8176
**CD14**	7.9[4.5–12]	2.2[0.8–7.3]	7.2[2.6–14]	3.3[0.5–15]	0.2673	0.6323
**CD11b^+^CD11c^+^**	2.5[0.8–7.5]	1.6[0.5–6.1]	4.0[1.0–8.6]	0.5[0.1–5.3]	0.7937	0.3690
**CD4^+^CD25^+^**	1.4[0.8–2.4]	2.8[1.8–4.2]	0.9[0.3–1.9]	0.7[0.1–3.6]	**0.0016**	0.0579
**T_regs_**	1.0[0.4–1.5]	2.3[1.4–3.1]	0.6[0.2–1.0]	0.5[0.0–2.3]	**0.0002**	**0.0291**
**T_regs_/T_eff_**	4.6[2.6–6.8]	4.9[2.9–8.0]	2.9[1.3–5.4]	3.5[0.4–7.6]	0.5774	0.3571
**CD4 N**	36[25–53]	21[12–37]	55[29–63]	19[4.8–52]	**0.0004**	0.8486
**CD4 CM**	27[15–42]	49[30–63]	25[14–31]	24[0.3–34]	**0.0166**	**0.0048**
**CD4 EM**	15[6.1–24]	13[4.5–20]	9.0[5.6–25]	8.0[0.3–22]	0.6158	0.2327
**CD4 TE**	7.8[5.3–11]	3.9[2.3–5.4]	8.1[6.4–21]	11[1.6–29]	0.1485	0.4839
**CD8 N**	34[21–54]	52[22–74]	31[13–56]	55[8.1–66]	**0.0173**	0.2722
**CD8 CM**	5.9[4.5–17]	14[2.8–27]	6.1[5.1–9.2]	7.8[1.0–31]	0.5531	0.4354
**CD8 EM**	7.0[2.9–14]	3.0[1.5–14]	4.8[1.2–17]	2.4[0.1–13]	0.0534	0.3395
**CD8 TE**	38[34–56]	12[6.4–26]	47[23–54]	14[4.4–35]	**0.0005**	0.8486
**CD4+CD127+**	61[39–83]	73[53–84]	73[41–93]	68[38–85]	**0.0175**	0.2713
**CD8+CD127+**	46[36–57]	67[56–83]	47[24–75]	60[29–76]	**0.0005**	0.1126
**CD4+CD38+**	2.2[1.4–9.4]	6.3[4.0–15]	1.3[1.1–7.4]	2.4[0.8–4.5]	**0.0143**	**0.0065**
**CD56+CD38+**	34[10–50]	17[7.2–50]	28[9.1–43]	4.1[2.8–9.0]	0.2634	**0.0031**

Values expressed as median[Interquartile range] of 30 subjects with pleural TB and 11 with pleural effusions caused by other diseases (non-TB); PB = peripheral blood; PF = pleural fluid; T_regs_ = regulatory T cells (CD4^+^CD25^+^CD127^lo^); T_eff_ = effector T cells (CD4+); N = naïve; CM = central memory; EM = effector memory; TE = terminal effector.

The proportion of CD56+ NK cells was significantly lower in the PF compared to the PB (median[IQR] = 2.5[1.6–4.6] and 8.6[4.6–13] respectively; p = 0.0001) but there was no difference in the proportion of B cells, γδ T cells, NKT cells or CD14+ monocytes. The proportion of CD4+CD25+ and CD4+CD25+CD127^lo^ T regulatory (T_reg_) cells were both significantly higher in the PF compared to the PB (p = 0.0016 and p = 0.0002 respectively). However, the ratio of T_reg_/T_eff_ cells did not change (median 4.6 for PB and 4.9 for PF; **Table 2**).

We next compared the PB subsets of TB and non-TB patients in order to see if PB markers could be used as a surrogate for pleural TB (**Table 2**). Interestingly, the proportion of CD4+ T cells was similar in TB and non-TB subjects in the blood (median[IQR] = 26[21–32] for TB and 22[15–27] for non-TB) but there was a significantly lower CD4∶CD8 ratio in TB patients (median = 1.6 for TB and 2.7 for non-TB; p = 0.0192) due to a higher (but not significant) proportion of CD8+ T cells (median[IQR] = 16[11–24] for TB and 8.4[4.8–17] for non-TB; **Table 2**). We also saw a significantly higher level of CD56+ NK cells in the PB of TB patients (median[IQR] = 8.6[4.6–13] compared to 5.3[3.5–9.0] for non-TB; p = 0.0296; **Table 2**). We did not see any differences in the total WBC levels, granulocyte, lymphocyte proportions (or ratio), MCV, hematocrit, hemoglobin or platelet levels (data not shown) between TB and non-TB subjects.

We next compared the PF subsets between TB and non-TB patients and found an increase in CD8+ T cells (median[IQR] = 17[12–23] for TB and 10[0.9–16] for non-TB (p = 0.0099)) but no difference in the proportion of CD4+ T cells (**Table 2**). However, within the CD4+ subset, there was a shift to a central memory phenotype with a median of 49% in TB compared to 23% in non-TB subjects (p = 0.0048). Despite the increase in proportion of CD8+ T cells, no difference in the proportions of naïve and memory subsets within the CD8+ T cell population was observed (**Table 2**). There was a 4-fold increase in the proportion of T_reg_ cells in the pleural fluid of TB patients (p = 0.0291) but again no difference in the ratio of T_reg_ to T_eff_ cells (**Table 2**). We also saw a significantly increased expression of CD38 on both the CD4+ (median% = 6.3 for TB and 2.4 for non-TB) and CD56+ cells (median% = 17 for TB and 4.1 for non-TB) (p = 0.0065 and p = 0.0031 respectively; **Table 2**). However, we saw no difference in the proportion of Ki-67 or perforin positive cells within the CD4+, CD8+ or CD56+ subsets (data not shown).

### Functional responses to TB-specific and non-specific stimuli are significantly higher in the PF compared to PB

**Figure 2:**
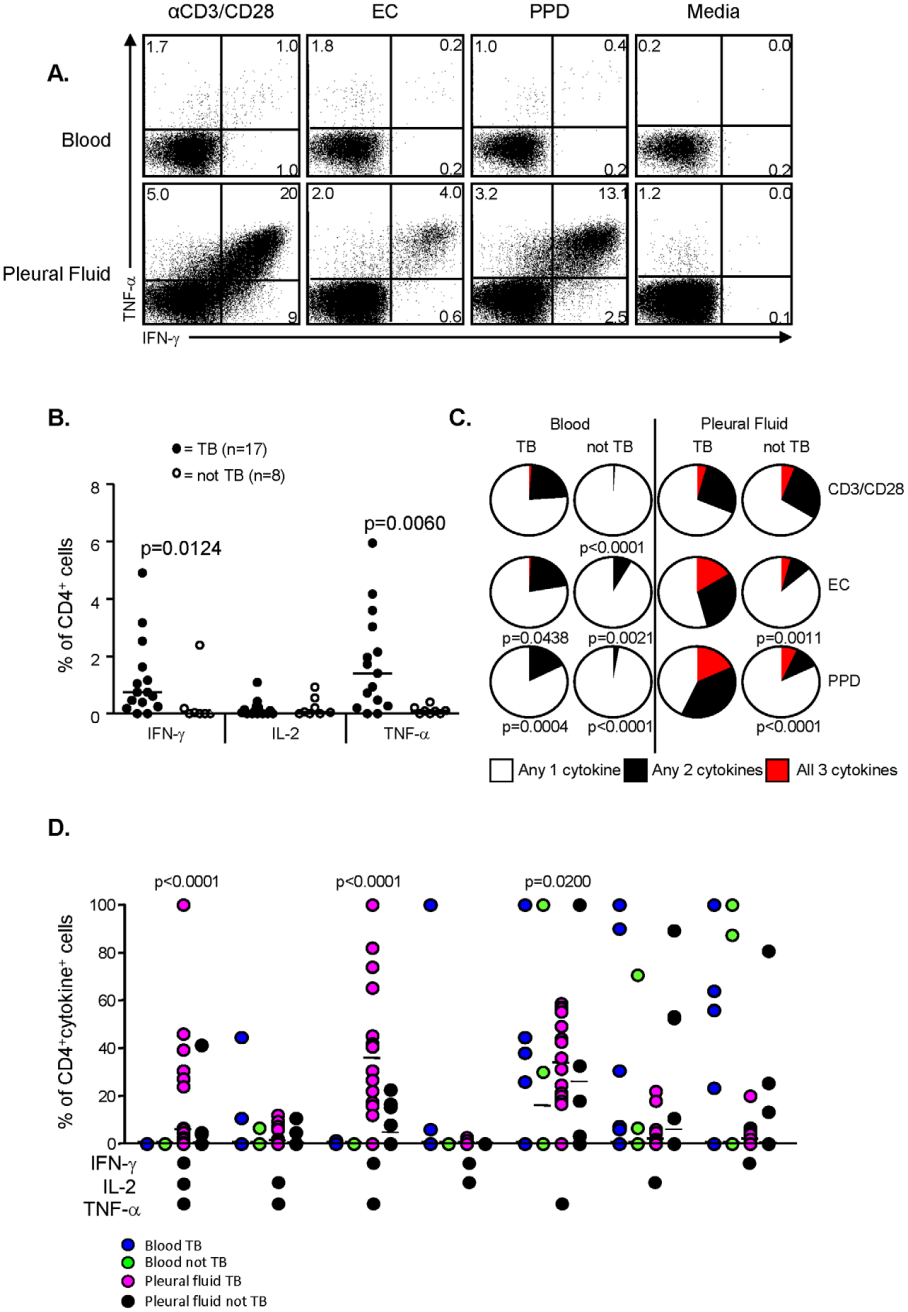
Quantitative and qualitative analysis of PPD-specific T cell responses. (**a**) Qualitative IFN-γ and TNF-α responses following overnight antigen stimulation of peripheral blood or pleural fluid cells. Shown are representative flow cytometry profiles from a patient with definite TB following overnight stimulation with anti-CD3/CD28 (positive control), ESAT-6/CFP-10 fusion protein (EC), PPD or unstimulated (media alone). (**b**) Quantitative assessment of IFN-γ, IL-2 and TNF-α secreting CD4+ T cells in subjects with TB and those without. Shown are the proportions of CD4+ T cells in the PF secreting IFN-γ, IL-2 or TNF-α in response to overnight stimulation with PPD. Statistical analysis was performed using a Mann-Whitney U test and p-values<0.05 were considered statistically significant (indicated). (**c**) Relative levels of polyfunctional T cell responses in subjects with TB or without. Shown are pie graphs demonstrating the proportion of cytokine-positive CD4+ T cells that produced only 1 of the cytokines (white), any 2 of the cytokines (black) or all 3 cytokines (red) from blood or pleural fluid of subjects with or without TB following anti-CD3/CD28, EC or PPD stimulation overnight. Statistical analysis of overall variance was performed using in-built SPICE software (ANOVA) and p-values indicate significantly decreased polyfunctionality compared to cells from the pleural fluid of subjects with TB. (**d**) Analysis of the functional profile of PPD-specific CD4+ T cells on the basis of simultaneous production of IFN-γ, IL-2 and TNF-α. All possible combinations of the 3 cytokines are shown along the y-axis. Statistical analysis was performed using a Mann-Whitney U- test and p-values are indicated (Pleural fluid TB significantly different to all 3 other groups).

The function of pleural and blood lymphocytes was evaluated by intracellular cytokine detection following overnight stimulation with TB-specific antigens (PPD and ESAT-6/CFP-10 (EC)) or positive control (αCD3/CD28 stimulation). Subjects classified with TB (probable or definite) had a distinct population of IFN-γ+TNF-α+ (IGTA) double-positive cells in the PF following stimulation with PPD and EC at much higher levels than the peripheral blood (**Fig. 1d** red box; **Fig. 2a**). Analysis of the PPD-IGTA population in the pleural fluid identified pleural TB with a sensitivity of 95% (95%CI = 75–100) and specificity of 100% (95%CI = 63–100) and was never seen in patients who did not have TB (negative predictive value of 100%). The memory phenotype of the IGTA population was also assessed based on CD27 and CD45RO expression (**Figs. 1e and 1f**). As expected, the majority of the responding cells were of an effector memory phenotype (CD27−CD45RO+; **Figs. 1e and 1f**) compared to naïve (CD27+CD45RO−) or central memory (CD27+CD45RO+) cells (p<0.001). Unexpectedly, the response in the PB was magnitudes lower than in the PF, even for the positive control stimulation (Fig. 2a). Furthermore, the quality of the response was also significantly different. Analysis of total IFN-γ, TNF-α or IL-2 producing cells showed a highly significant increase in the proportion of CD4+IFN-γ+ and CD4+TNF-α+ cells in TB patients compared to non-TB (p = 0.0124 and p = 0.0060 respectively; **Fig. 2b**). Analysis of the combinatorial responses showed a significant increase in the proportion of triple positive (IFN-γ+IL-2+TNF-α+) and double positive (TNF-α+IFN-γ+) cells in TB compared to non-TB patients following both PPD and EC stimulation (p = 0.0011 and p<0.0001 respectively; **Figs. 2c and 2d**). This was only seen in the PF and not the PB where the majority of responses were single-positive cytokines (**Figs. 2c and 2d**).

### Soluble biomarkers for pleural TB classification

**Figure 3:**
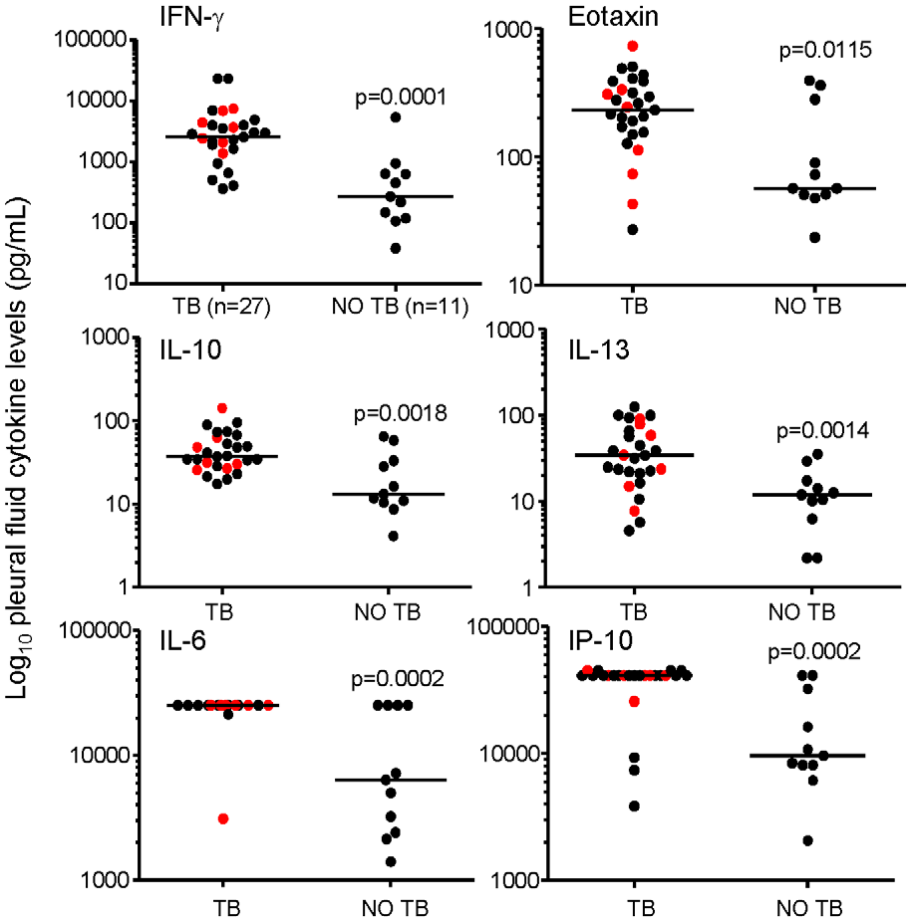
Multiplex cytokine analysis of ex vivo pleural fluid samples. Pleural fluid from subjects with TB (n = 27) and subjects without TB (n = 11) was assessed for 27 different cytokines using a Bioplex multi-cytokine analyser. Results are shown for IFN-γ, Eotaxin, IL-10, IL-13, IL6 and IP-10 levels in unstimulated fluid. Statistical analysis was performed using logistic regression analysis with Stata 3 software and multiple comparisons corrected for using Bonferonni's correction. Significance was set at p<0.05 and are indicated. Red dots indicate HIV-positive subjects.

We performed 27-plex cytokine analysis on unstimulated pleural fluid to determine which marker(s) could best predict pleural TB. A high level of IFN-γ has previously been seen in un-manipulated pleural fluid samples from TB patients in South Africa [16]. In the present study IFN-γ levels >1171 pg/mL resulted in a high degree of sensitivity and specificity and 88% correct classification of pleural effusions caused by TB or not (p = 0.0003; **Fig. 3** **and** **Table 3**). We also found a number of other markers that accurately discriminated between TB and non-TB in unstimulated pleural fluid. These included significantly higher levels of Eotaxin (p = 0.0243), IL-10 (p = 0.0034), IL-13 (p = 0.0025), IL-6 (p = 0.0005) and IP-10 (p = 0.0004) in subjects with TB compared to those without (**Fig. 3**). Logistic regression analysis showed that levels of IP-10 >36,695 pg/mL could discriminate between subjects with TB and those without with a specificity of 82% and sensitivity of 85% (AUC of 0.84) (**Fig. 3** **and** **Table 3**). This was slightly lower than IFN-γ but IP-10 resulted in the best likelihood ratio (positive and negative = 9.6 and 0.1 respectively compared to positive LR of 2.8 for IFN-γ; data not shown). Levels of IL-6 >23,254 pg/mL resulted in a sensitivity of 93% but a comparatively low specificity (64%; **Table 3**). IL-10 was 100% sensitive but again, low in specificity (64%) at levels >17 pg/mL. Multivariate analysis showed that a combination of IL-6, IL-10 and IP-10 increased the AUC to 0.96 (**Table 3**), with the positive likelihood ratio increasing to 10.

**Table 3:** Sensitivity and specificity of unstimulated pleural fluid cytokine levels.

parameter	sensitivity	specificity	AUC	cut-off (pg/mL)
**IFN-γ**	81	91	0.88	>1171
**Eotaxin**	73	89	0.74	<102
**IL-10**	100	64	0.81	>17
**IL-13**	78	82	0.82	>19
**IL-6**	93	64	0.79	>23254
**IP-10**	85	82	0.84	>36695
**IP-10+IL-6+ IL-10**	92	91	0.96	not applicable

## Discussion

In this study we show that the immune profile in the pleural fluid is distinct from the peripheral blood in subjects with pleural TB illustrating the sequestration of effector cells to the site of infection at this acute stage of disease. Importantly, we have determined both cellular and soluble biomarkers in the pleural fluid that can easily discriminate between pleural effusions caused by TB and those caused by other diseases such as pneumonia or malignancy. These findings provide valuable insight into TB pathogenesis and, once validated in a larger cohort, will aid in the development of new and improved TB diagnostics.

The first aim of this study was to determine if the peripheral blood is representative of what is occurring in the lung during pleural TB. We found it was very distinct presumably reflecting of the sequestration of CD4+ effector cells to the lungs during this acute stage of disease. Indeed, the proportion of CD4+ T cells in the pleural fluid was significantly increased in TB patients and had a memory phenotype compared to the blood of the same patients or blood and pleural fluid of the non-TB patients. T_reg_ cells were also increased in the pleural fluid compared to blood of TB patients however this was synchronous with the increase in effector CD4+ T cells such that the ratio of T_reg_ to T_eff_ cells was not significantly different; in keeping with their immunomodulatory role in protecting against excessive inflammation that is associated with an increase in effector T cells. This finding suggests that T_reg_ increases should always be assessed alongside CD4+ T_eff_ cells. The second aim of this study was to determine if peripheral blood cells could provide a surrogate biomarker for pleural TB if pleural effusions could not be obtained. However, having analysed over 40 subsets of cells, the only differences we saw between TB and non-TB patients were an increase in CD8+ T cells and CD56+ NK cells; most likely non-specific.

Supporting our findings on the response of cells to antigen stimulation, a recent paper showed high levels of polyfunctional T cells with an effector memory phenotype in subjects with pleural TB [10], whilst PPD-specific responses in the BAL have been shown to be significantly higher in pulmonary and extrapulmonary TB patients compared to non-tuberculous control subjects [12]. However, few studies have directly compared the blood and lung responses from the same subjects. Surprisingly, we saw a significantly lower response to antigenic stimulation from peripheral blood cells compared to the pleural fluid cells, presumably due to sequestration of the effector memory population to the site of infection. Indeed, analysis of the PPD-responsive cells in the pleural fluid resulted in 95% correct classification of TB disease or not. Cellular analysis of the pleural effusions also allowed us to diagnose other underlying causes, particularly malignancies, and was also applicable for analysis of ascites fluid. Our results indicate that use of blood-based diagnostics may be inaccurate at this acute stage of disease and possibly accounts for the reduced sensitivity for active TB disease with the current IFN-γ release assays (IGRAs) [17]. We believe flow cytometry should be included in a diagnostic algorithm for extrapulmonary TB where feasible; however its use as a rapid diagnostic test is limited in resource-poor settings.

Therefore, the final aim of this study was to determine soluble biomarkers for pleural TB, which presents the most viable option for development of a field-friendly, rapid diagnostic test. Previous studies have found high levels of IFN-γ in *ex vivo* pleural fluid [16] with a recent meta-analysis showing close to 100% specificity and sensitivity for pleural TB [18]. However, we found much lower sensitivity using IFN-γ alone in our study despite the relatively high levels in unstimulated fluid, presumably reflecting genetic differences in the study sites. Alongside IFN-γ, we found high levels of IP-10 and IL-6 with a combination of IP-10, IL-6 and IL-10 resulting in 96% correct classification of pleural TB. IP-10 is primarily (but not solely) induced by IFN-γ (with enhanced production seen with dual TNF-α and IFN-γ stimulation [19]) and is a potent chemo-attractant for activated T cells [19]. It has previously been shown to be elevated in tuberculous pleurisy [19], [20], although the discriminatory power compared to IFN-γ for both pulmonary and pleural TB appears to be variable [19]–[23]. IL-6 has also been shown to be increased in pleural TB [11], is important in the pro-inflammatory response and has recently been shown to be one of the most important biomarkers in TB, alongside IP-10 and IL-10 [24].

In conclusion, this study defines both cellular and soluble biomarkers that provide high levels of sensitivity and specificity for pleural TB in a West African cohort. Whilst cellular biomarkers are currently not applicable for generation of a rapid diagnostic test, the use of flow cytometry as part of the diagnostic algorithm for extrapulmonary TB is important where feasible. However, a combination of soluble biomarkers (IP-10, IL-10 and IL-6), resulted in high specificity and sensitivity for pleural TB, were not affected by HIV status and, once validated, hold great promise for development of a rapid diagnostic test for pleural TB.
